# Antimycobacterial activity of nitrogen heterocycles derivatives: 7-(pyridine-4-yl)-indolizine derivatives. Part VII^8–12^

**DOI:** 10.1080/14756366.2017.1375483

**Published:** 2017-10-26

**Authors:** Anda-Mihaela Olaru, Violeta Vasilache, Ramona Danac, Ionel I. Mangalagiu

**Affiliations:** a Faculty of Chemistry, “Alexandru Ioan Cuza” University of Iasi, Iasi, Romania;; b “Alexandru Ioan Cuza” University of Iasi, CERNESIM Research Centre, Iasi, Romania

**Keywords:** Antimycobacterial, indolizine, pyridinium salts, ADMET

## Abstract

A series of 13 compounds having a monoindolizine mono-salt skeleton was designed and synthesised in order to evaluate their antimycobacterial activity. The synthesis is efficient, involving only three steps: two alkylations and one 3 + 2 dipolar cycloaddition. The antimicrobial activity against *Mycobacterium tuberculosis* H37Rv grown under aerobic conditions was evaluated, eight compounds showing a very good antimycobacterial activity. SAR correlation reveals a certain influence of the R substituent from the *para* position of benzoyl moiety at position 3 of indolizine. The most active five compounds passed the second stage of anti-TB testing, the assay demonstrating that they are potent against both replicating and non-replicating *Mtb*, have a bactericidal mechanism of action, are active against drug-resistant *Mtb* strains, present a moderate to good activity against nontuberculous mycobacteria, a good intracellular activity, and a moderate to high cytotoxicity. For one compound showing a promising anti-TB profile, a complete ADMET study has been performed.

## Introduction

Tuberculosis is one of the major causes of disability and death worldwide^1^, remaining one of the top 10 causes of deaths in 2015^2^. More than 95% of TB deaths occur in low- and middle-income countries, according to the World Health Organisation^2^. In 2015, 10.4 million people became ill with TB, and 1.4 million people died from the disease. An additional 0.4 million deaths resulted from TB disease among people living with HIV^2^. Globally in 2015, an estimated 480,000 people developed multidrug-resistant TB (MDR-TB) and around 100,000 people developed rifampicin-resistant TB^2^. The emergence of MDR-TB and, more recently, extensively drug-resistant (XDR) TB has intensified the need for new TB drugs. Major efforts are done for the discovery and development of new TB drug targets and candidate drugs, and evaluation of novel TB drugs and optimal drug combinations in preclinical and clinical studies^2^^,^^3^. There are currently two important strategies used for discovery of new anti-TB drugs^4^^,^^5^. One involves the synthesis of analogous of the existing drugs, and the other refers to the search for novel structures. Between the various classes of organic compounds, fused N-heterocyles, especially pyridine fused systems, showed promising anti-TB activity against replicating *Mycobacterium tuberculosis* (*Mtb*) H37Rv, similar to isoniazid^6^^,^^7^.

In our previous work, we showed that several new classes of compounds with fused heterocyclic structure possess antimicrobial activity^8–14^, antimycobacterial including^8–12^. Recently, we reported compound **1** with monoindolizine mono-pyridinium salt structure showing a promising antimycobacterial activity against both replicating and non-replicating *Mtb*^8^.

These results prompted us to extend our study to a series of compounds having the same monoindolizine mono-pyridinium skeleton, but different substituents on adjacent phenyl rings, in order to have a better understanding of the acting mode of these compounds and to be able to see any influence the substituents have on the antimycobacterial activity. The results presented herein refer to the synthesis and antimycobacterial evaluation of a new series of thirteen compounds.

## Methods

### General

Melting points were recorded on a A. Krüss Optronic Melting Point Meter KSPI and are uncorrected. Proton and carbon nuclear magnetic resonance (*δ*_H_, *δ*_C_) spectra were recorded on a DRX-500 Bruker (Bruker, Bremen, Germany) (500 MHz). All chemical shifts are quoted on the *δ*-scale in ppm. Coupling constants are given in Hz. IR spectra were recorded on a FTIR Shimadzu spectrometer. Thin layer chromatography (TLC) was carried out on Merck silica gel 60F_254_ plates. Visualisation of the plates was achieved using a UV lamp (*λ*_max_ =  254 or 365 nm).

### *General procedure for synthesis of quaternary salts* 6a–m

The monoindolizine **5** (1 mmol, 1 equiv., 0.37 g **5a**, 0.40 g **5 b**, 0.45 g **5c**, 0.38 g **5d**, 0.40 g **5e**) and bromacetophenone derivative (*p* or/and *m* substituted, 2 mmol, 2 equiv.) was suspended in anhydrous acetone (20 ml) and magnetically stirred over night at reflux. The resulting precipitate was collected by filtration and then washed with acetone. All products were purified by crystallisation (CHCl_3_:MeOH 1:1, v:v).

*4*-*(3-Benzoyl-1-(ethoxycarbonyl)indolizine-7-yl)-1-(2-oxo-2-(p-tolyl)ethyl)pyridin-1-ium bromide* (**6a**). Orange powder (0.52 g, 89% yield), mp = 279–282 °C. ^1^H-NMR (400 MHz, DMSO-d_6_): *δ* 1.36 (t, *J* = 7.2 Hz, 3H, H_12_), 2.47 (s, 3H, CH_3_), 4.37 (q, *J* = 7.2 Hz, 2H, H_11_), 6.53 (s, 2H, H_22_), 7.50 (d, *J* = 8.0 Hz, 2H, H_26_, H_28_), 7.63 (t, *J* = 7.2 Hz, 2H, 2 × H_16_), 7.70 (s, 1H, H_2_), 7.71 (t, *J* = 7.2 Hz, 1H, H_17_), 7.84 (d, *J* = 7.2 Hz, 2H, 2 x H_15_), 7.96 (dd, *J* = 7.6 Hz, *J* = 1.6 Hz, 1H, H_6_), 8.01 (d, *J* = 8.0 Hz, 2H, H_25_, H_29_), 8.79 (d, *J* = 6.8 Hz, 2H, 2 × H_19_), 8.93 (as, 1H, H_8_), 9.14 (d, *J* = 6.8 Hz, 2H, 2 × H_20_), 9.92 (d, *J* = 7.6 Hz, 1H, H_5_). ^13 ^C-NMR (125 MHz, DMSO-d_6_): *δ* 14.3 C_12_, 21.3 CH_3_, 60.1 C_11_, 65.5 C_22_, 108.1 C_1_, 113.7 C_6_, 118.8 C_8_, 123.0 C_3_, 124.6 2 × C_19_, 127.9 C_2_, 128.3 C_25_, C_29_, 128.6 2 × C_15_, 128.7 2 × C_16_, 129.1 C_5_, 129.6 C_26_, C_28_, 131.0 C_24_, 132.1 C_17_, C_7_, 137.8 C_9_, 138.7 C_14_, 145.4 C_27_, 146.6 2 × C_20_, 152.3 C_18_, 162.6 C_10_, 184.9 C_13_, 190.1 C_23_. IR (KBr, *ν*(cm^−1^): 3399, 3032, 3974, 1707, 1643, 1622, 1642, 1205. Anal. Calcd. for C_32_H_27_BrN_2_O_4_: C, 65.87; H, 4.54; N, 4.66; Found: C, 65.93; H, 4.50; N, 4.75.

*4-(3-Benzoyl-1-(ethoxycarbonyl)indolizine-7-yl)-1-(2-(3-methoxyphenyl)-2-oxoethyl)pyridin-1-ium* (**6b**). Yellow powder (0.53 g, 89% yield), mp 255–256 °C. ^1^H-NMR (400 MHz, DMSO-d_6_): *δ* 1.37 (t, *J* = 7.2 Hz, 3H, H_12_), 3.89 (s, 3H, OCH_3_), 4.37 (q, *J* = 7.2 Hz, 2H, H_11_), 6.58 (s, 2H, H_22_), 7.40 (dd, *J* = 8.4 Hz, *J* = 2.4 Hz, 1H, H_27_), 7.59–7.64 (m, 4H, H_26_, H_29_, 2 × H_16_), 7.68–7.73 (m, 3H, H_2_, H_17_, H_25_), 7.84 (d, *J* = 7.2 Hz, 2H, 2 × H_15_), 7.96 (dd, *J* = 7.6 Hz, *J* = 1.6 Hz, 1H, H_6_), 8.80 (d, *J* = 6.8 Hz, 2H, 2 × H_19_), 8.92 (as, 1H, H_8_), 9.15 (d, *J* = 6.8 Hz, 2H, 2 × H_20_), 9.90 (d, *J* = 7.6 Hz, 1H, H_5_). ^13^C-NMR (125 MHz, DMSO-d_6_): *δ* 14.2 C_12_, 55.6 OCH_3_, 60.1 C_11_, 65.7 C_22_, 108.1 C_1_, 112.9 C_29_, 113.6 C_6_, 118.8 C_8_, 120.5 C_27_, 120.6 C_25_, 123.0 C_3_, 124.6 2 × C_19_, 127.8 C_2_, 128.6 2 × C_15_, 128.7 2 × C_16_, 129.1 C_5_, 130.4 C_26_, 134.8 C_24_, 132.0 C_17_, 132.1 C_7_, 137.7 C_9_, 138.6 C_14_, 146.5 2 × C_20_, 152.2 C_18_, 159.5 C_28_, 162.6 C_10_, 184.9 C_13_, 190.6 C_23_. IR (KBr, *ν*(cm^−1^): 3032, 2976, 1697, 1624, 1342, 1198. Anal. Calcd. for C_32_H_27_BrN_2_O_5_: C, 64.11; H, 4.54; N, 4.67; Found: C, 64.23; H, 4.45; N, 4.70.

*4-[3-(4-Chlorophenyl)-1-(ethoxycarbonyl)indolizine-7-yl)-1-(2-(4-methoxyphenyl)-2-oxoethyl)pyridine-1-ium bromide* (**6c**). Yellow powder (0.59 g, 93% yield), mp 288 °C. ^1^H-NMR (400 MHz, DMSO-d_6_): *δ* 1.36 (t, *J* = 7.2 Hz, 3H, H_12_), 3.91 (s, 3H, OCH_3_), 4.37 (q, *J* = 7.2 Hz, 2H, H_11_), 6.49 (s, 2H, H_22_), 7.20 (d, *J* = 8.8 Hz, 2H, H_26_, H_28_), 7.68 (d, *J* = 8.4 Hz, 2H, 2 × H_16_), 7.73 (s, 1H, H_2_), 7.86 (d, *J* = 8.4 Hz, 2H, 2 × H_15_), 7.96 (dd, *J* = 7.2 Hz, *J* = 2.0 Hz, 1H, H_6_), 8.08 (d, *J* = 8.8 Hz, 2H, H_25_, H_29_), 8.78 (d, *J* = 7.2 Hz, 2H, 2 × H_19_), 8.92 (d, *J* = 1.2 Hz, 1H, H_8_), 9.12 (d, *J* = 6.8 Hz, 2H, 2 × H_20_), 9.88 (d, *J* = 7.2 Hz, 1H, H_5_). ^13 ^C-NMR (125 MHz, DMSO-d_6_): *δ* 14.3 C_12_, 55.9 OCH_3_, 60.3 C_11_, 65.4 C_22_, 108.3 C_1_, 113.9 C_6_, 114.5 C_26_, C_28_, 118.9 C_8_, 122.9 C_3_, 124.7 2 × C_19_, 126.3 C_24_, 128.0 C_2_, 128.8 2 × C_16_, 129.3 C_5_, 130.8 2 × C_15_, C_25_, C_29_, 132.3 C_7_, 137.0 C_17_, 137.4 C_14_, 138.0 C_9_, 146.7 2 × C_20_, 152.3 C_18_, 162.7 C_10_, 164.3 C_27_, 183.7 C_13_, 189.0 C_23_. IR (KBr, *ν*(cm^−1^): 3395, 3022, 2936, 1707, 1680, 1642, 1242, 1206, 1173. Anal. Calcd. for C_32_H_26_BrClN_2_O_5_: C, 60.63; H, 4.13; N, 4.42; Found: C, 60.70; H, 4.10; N, 4.45.

*4-(3-(4-Chlorobenzoyl)-1-(ethoxycarbonyl)indolizine-7-yl)-1-(2-(3-methoxyphenyl)-2-oxoethyl)pyridin-1-ium* (**6d**). Yellow powder (0.46 g, 73% yield), mp 252–254 °C. ^1^H-NMR (400 MHz, DMSO-d_6_): *δ* 1.36 (t, *J* = 7.2 Hz, 3H, H_12_), 3.88 (s, 3H, OCH_3_), 4.37 (q, *J* = 7.2 Hz, 2H, H_11_), 6.52 (s, 2H, H_22_), 7.40 (dd, *J* = 8.4 Hz, *J* = 2.4 Hz, 1H, H_27_), 7.58 (as, 1H, H_29_), 7.62 (t, *J* = 8.0 Hz, 1H, H_26_), 7.68–7.71 (m, 3H, 2 × H_16_, H_25_), 7.75 (s, 1H, H_2_), 7.87 (d, *J* = 8.4 Hz, 2H, 2 × H_15_), 7.97 (dd, *J* = 7.6 Hz, *J* = 2.0 Hz, 1H, H_6_), 8.80 (d, *J* = 6.8 Hz, 2H, 2 × H_19_), 8.92 (d, *J* = 0.8 Hz, 1H, H_8_), 9.11 (d, *J* = 6.8 Hz, 2H, 2 × H_20_), 9.91 (d, *J* = 7.2 Hz, 1H, H_5_). ^13 ^C-NMR (125 MHz, DMSO-d_6_): *δ* 14.3 C_12_, 55.6 OCH_3_, 60.2 C_11_, 65.8 C_22_, 108.3 C_1_, 113.0 C_29_, 113.8 C_6_, 118.9 C_8_, 120.5 C_25_, 120.7 C_27_, 123.0 C_3_, 124.7 2 × C_19_, 128.0 C_2_, 128.8 2 × C_16_, 129.3 C_5_, 130.4 C_26_, 130.7 2 × C_15_, 132.3 C_7_, 134.9 C_24_, 137.0 C_17_, 137.0 C_14_, 138.0 C_9_, 146.6 2 × C_20_, 152.4 C_18_, 159.6 C_28_, 162.7 C_10_, 183.7 C_13_, 190.6 C_23_. IR (KBr, *ν*(cm^−1^): 3030, 2920, 1701, 1643, 1248, 1198. Anal. Calcd. for C_32_H_26_BrClN_2_O_5_: C, 60.63; H, 4.13; N, 4.42; Found: C, 60.65; H, 4.10; N, 4.48.

*4*-(*3-(4-Chlorobenzoyl)-1-(ethoxycarbonyl)indolizine-7-yl)-1-(2-(4-fluorophenyl)-2-oxoethyl)pyridin-1-ium bromide* (**6e**). Orange powder (0.55 g, 89% yield), mp 307–310 °C. ^1^H-NMR (400 MHz, DMSO-d_6_): *δ* 1.37 (t, *J* = 7.2 Hz, 3H, H_12_), 4.38 (q, *J* = 7.2 Hz, 2H, H_11_), 6.51 (s, 2H, H_22_), 7.55 (t, *J* = 8.8 Hz, 2H, H_26_, H_28_), 7.70 (d, *J* = 8.4 Hz, 2H, 2 × H_16_), 7.76 (s, 1H, H_2_), 7.88 (d, *J* = 8.4 Hz, 2H, 2 × H_15_), 7.98 (ad, *J* = 7.2 Hz, 1H, H_6_), 8.20 (dd, *J* = 8.4 Hz, *J* = 5.6 Hz, 2H, H_25_, H_29_), 8.81 (d, *J* = 6.4 Hz, 2H, 2 × H_19_), 8.97 (as, 1H, H_8_), 9.11 (d, *J* = 6.4 Hz, 2H, 2 × H_20_), 9.92 (d, *J* = 7.2 Hz, 1H, H_5_). ^13^C-NMR (125 MHz, DMSO-d_6_): *δ* 14.3 C_12_, 60.2 C_11_, 65.5 C_22_, 108.2 C_1_, 113.8 C_6_, 116.3 (d, C_26_, C_28_, J= 22 Hz), 118.8 C_8_, 122.9 C_3_, 124.7 2 × C_19_, 127.9 C_2_, 128.7 2 × C_16_, 129.2 C_5_, 130.3 (d, C_24_, J= 3.0 Hz), 130.7 2 × C_15_, 131.4 (d, C_25_, C_29_, J= 10.0 Hz), 132.2 C_7_, 137.0 C_17_, 137.3 C_14_, 137.9 C_9_, 146.6 2 × C_20_, 152.3 C_18_, 162.6 C_10_, 165.7 (d, C_27_, J= 253 Hz), 183.6 C_13_, 189.4 C_23_. IR (KBr, *ν*(cm^−1^): 3024, 3926, 1713, 1624, 1348, 1204. Anal. Calcd. for C_31_H_23_BrClFN_2_O_4_: C, 59.87; H, 3.73; N, 4.50; Found: C, 59.93; H, 3.70; N, 4.54.

*4-(3-(4-Bromobenzoyl)-1-(ethoxycarbonyl)indolizine-7-yl)-1-(2-oxo-2-(p-tolyl)ethyl)pyridin-1-ium bromide* (**6f**). Orange powder (0.61 g, 92% yield), mp 283–284 °C. ^1^H-NMR (400 MHz, DMSO-d_6_): *δ* 1.37 (t, *J* = 7.2 Hz, 3H, H_12_), 2.47 (s, 3H, CH_3_), 4.37 (q, *J* = 7.2 Hz, 2H, H_11_), 6.51 (s, 2H, H_22_), 7.50 (d, *J* = 8.0 Hz, 2H, H_26_, H_28_), 7.74 (s, 1H, H_2_), 7.79 (d, *J* = 8.4 Hz, 2H, 2 × H_16_), 7.83 (d, *J* = 8.4 Hz, 2H, 2 × H_15_), 7.96 (ad, *J* = 7.6 Hz, 1H, H_6_), 8.01 (d, *J* = 8.0 Hz, 2H, H_25_, H_29_), 8.78 (d, *J* = 7.2 Hz, 2H, 2 × H_19_), 8.94 (as, 1H, H_8_), 9.12 (d, *J* = 6.4 Hz, 2H, 2 × H_20_), 9.90 (d, *J* = 7.2 Hz, 1H, H_5_). ^13 ^C-NMR (125 MHz, DMSO-d_6_): *δ* 14.4 C_12_, 21.4 CH_3_, 60.3 C_11_, 65.6 C_22_, 108.3 C_1_, 113.9 C_6_, 118.9 C_8_, 122.9 C_3_, 124.7 2 × C_19_, 126.1 C_17_, 128.1 C_2_, 128.4 C_25_, C_29_, 129.3 C_5_, 129.7 C_26_, C_28_, 130.9 2 × C_16_, 131.1 C_24_, 131.8 2 × C_15_, 132.4 C_7_, 137.8 C_14_, 138.0 C_9_, 145.6 C_27_, 146.7 2 × C_20_, 152.4 C_18_, 162.7 C_10_, 183.9 C_13_, 190.2 C_23_. IR (KBr, *ν*(cm^−1^): 3419, 3021, 2930, 1705, 1682, 1622, 1344, 1206. Anal. Calcd. for C_32_H_26_Br_2_N_2_O_4_: C, 58.03; H, 3.96; N, 4.23; Found: C, 58.07; H, 3.93; N, 4.25.

*4-[3-(4-Bromophenyl)-1-(ethoxycarbonyl)indolizine-7-yl)-1-(2-(4-methoxyphenyl)-2-oxoethyl)pyridine-1-ium bromide* (**6g**). Orange powder (0.56 g, 82% yield), mp 277–278 °C. ^1^H-NMR (400 MHz, DMSO-d_6_): *δ* 1.36 (t, *J* = 7.2 Hz, 3H, H_12_), 3.92 (s, 3H, OCH_3_), 4.37 (q, *J* = 7.2 Hz, 2H, H_11_), 6.45 (s, 2H, H_22_), 7.20 (d, *J* = 8.8 Hz, 2H, H_26_, H_28_), 7.76 (s, 1H, H_2_), 7.79 (d, *J* = 8.4 Hz, 2H, 2 × H_16_), 7.84 (d, *J* = 8.4 Hz, 2H, 2 × H_15_), 7.97 (dd, *J* = 7.2 Hz, *J* = 1.6 Hz, 1H, H_6_), 8.08 (d, *J* = 8.8 Hz, 2H, H_25_, H_29_), 8.78 (d, *J* = 7.2 Hz, 2H, 2 × H_19_), 8.96 (as, 1H, H_8_), 9.10 (d, *J* = 6.8 Hz, 2H, 2 × H_20_), 9.92 (d, *J* = 7.2 Hz, 1H, H_5_). ^13 ^C-NMR (125 MHz, DMSO-d_6_): *δ* 14.3 C_12_, 55.8 OCH_3_, 60.2 C_11_, 65.3 C_22_, 108.2 C_1_, 113.8 C_6_, 114.4 C_26_, C_28_, 118.8 C_8_, 122.8 C_3_, 124.9 2 × C_19_, 126.0 C_17_, 126.2 C_24_, 127.9 C_2_, 129.2 C_5_, 130.7 C_25_, C_29_, 130.8 2 × C_16_, 131.6 2 × C_15_, 132.2 C_7_, 137.6 C_14_, 137.9 C_9_, 146.6 2 × C_20_, 152.2 C_18_, 162.6 C_10_, 164.2 C_27_, 183.7 C_13_, 188.9 C_23_. IR (KBr, *ν*(cm^−1^): 3406, 3018, 2932, 1713, 1680, 1622, 1346, 1205. Anal. Calcd. for C_32_H_26_Br_2_N_2_O_5_: C, 56.66; H, 3.86; N, 4.13; Found: C, 56.69; H, 3.85; N, 4.16.

*4-(3-(4-Bromobenzoyl)-1-(ethoxycarbonyl)indolizine-7-yl)-1-(2-(3-methoxyphenyl)-2-oxoethyl)pyridin-1-ium* (**6h**). Yellow powder (0.67 g, 99% yield), mp 256–259 °C. ^1^H-NMR (400 MHz, DMSO-d_6_): *δ* 1.37 (t, *J* = 7.2 Hz, 3H, H_12_), 3.89 (s, 3H, OCH_3_), 4.38 (q, *J* = 7.2 Hz, 2H, H_11_), 6.55 (s, 2H, H_22_), 7.40 (dd, *J* = 8.4 Hz, *J* = 2.4 Hz, 1H, H_27_), 7.59 (as, 1H, H_29_), 7.62 (t, *J* = 8.0 Hz, 1H, H_26_), 7.70–7.74 (m, 2H, H_2_, H_25_), 7.79 (d, *J* = 8.4 Hz, 2H, 2 × H_16_), 7.83 (d, *J* = 8.4 Hz, 2H, 2 × H_15_), 7.97 (dd, *J* = 7.6 Hz, *J* = 2.0 Hz, 1H, H_6_), 8.80 (d, *J* = 6.8 Hz, 2H, 2 × H_19_), 8.94 (d, *J* = 1.2 Hz, 1H, H_8_), 9.13 (d, *J* = 6.8 Hz, 2H, 2 × H_20_), 9.89 (d, *J* = 7.6 Hz, 1H, H_5_). ^13 ^C-NMR (125 MHz, DMSO-d_6_): *δ* 14.3 C_12_, 55.6 OCH_3_, 60.2 C_11_, 65.8 C_22_, 108.3 C_1_, 113.0 C_29_, 113.8 C_6_, 118.8 C_8_, 120.5 C_27_, 120.7 C_25_, 122.9 C_3_, 124.7 2 × C_19_, 126.0 C_17_, 128.0 C_2_, 129.2 C_5_, 130.4 C_26_, 130.8 2 × C_16_, 131.7 2 × C_15_, 132.3 C_7_, 134.8 C_24_, 137.7 C_14_, 137.9 C_9_, 146.6 2 × C_20_, 152.3 C_18_, 159.6 C_28_, 162.7 C_10_, 183.8 C_13_, 190.6 C_23_. IR (KBr, *ν*(cm^−1^): 3025, 2930, 1701, 1642, 1248, 1200, 1171. Anal. Calcd. for C_32_H_26_Br_2_N_2_O_5_: C, 56.66; H, 3.86; N, 4.13; Found: C, 56.68; H, 3.83; N, 4.16.

*4-(3-(4-Chlorobenzoyl)-1-(ethoxycarbonyl)indolizine-7-yl)-1-(2-(2,4-hydroxyphenyl)-2-oxoethyl)pyridin-1-ium* (**6m**). Yellow powder (0.35 g, 56% yield), mp 265–267 °C. ^1^H-NMR (400 MHz, DMSO-d_6_): *δ* 1.37 (t, *J* = 7.2 Hz, 3H, H_12_), 4.37 (q, *J* = 7.2 H, 2H, H_11_z), 6.43 (s, 2H, H_22_), 7.02 (d, *J* = 8.4 Hz, 1H, H_28_), 7.49 (s, 1H, H_25_), 7.52 (d, *J* = 8.4 Hz, 1H, H_29_), 7.69 (d, *J* = 8.0 Hz, 2H, 2 × H_16_), 7.73 (s, 1H, H_2_), 7.87 (d, *J* = 8.0 Hz, 2H, 2 × H_15_), 7.96 (d, *J* = 7.2 Hz, 1H, H_6_), 8.76 (d, *J* = 6.0 Hz, 2H, 2 × H_19_), 8.93 (s, 1H, H_8_), 9.12 (d, *J* = 6.0 Hz, 2H, 2 × H_20_), 9.89 (d, *J* = 7.2 Hz, 1H, H_5_), 9.70 (s, 1H, OH), 10.41 (s, 1H, OH). ^13^C-NMR (125 MHz, DMSO-d_6_): *δ* 14.3 C_12_, 60.2 C_11_, 65.2 C_22_, 108.2 C_1_, 115.0 C_25_, 115.5 C_28_, 113.8 C_6_, 118.8 C_8_, 121.8 C_29_, 122.9 C_3_, 124.6 2 × C_19_, 125.1 C_24_, 128.0 C_2_, 128.8 2 × C_16_, 129.2 C_5_, 130.7 2 × C_15_, 132.3 C_7_, 137.0 C_17_, 137.4 C_14_, 137.9 C_9_, 146.6 2 × C_20_, 145.7 C_26_, 152.1 C_18_, 152.3 C_27_, 162.7 C_10_, 183.7 C_13_, 189.6 C_23_. IR (KBr, *ν*(cm^−1^): 3420, 3030, 2974, 1693, 1609, 1526, 1204, 1084. Anal. Calcd. for C_31_H_24_Cl_2_N_2_O_6_: C, 62.95; H, 4.09; N, 4.74; Found: C, 62.98; H, 4.03; N, 4.76.

### Microbiology

Compounds were evaluated for antimycobacterial activity against *M. Tuberculosis*, as a part of the TAACFTB screening program under direction of the US National Institute of Health, the NIAID division. Antimycobacterial activities of the compounds were performed by Center of Tuberculosis Antimicrobial Acquisition and Coordinating Facility (TAACF) at Southern Research Institute. All protocols concerning the antimycobacterial evaluation of tested compounds can be found in the Supplementary Appendix.

## Results and discussion

### Design and synthesis

Our strategy included the synthesis of compounds with the same 4-(indolizine-7-yl)-pyridin-1-ium scaffold as in compound **1**, but with various substituents on both phenyl rings. Having in mind the observation that a (*p*)substituted-benzoyl moiety is usefully pharmacophoric unit for the antimycobacterial activity^10^^,^^12^^,^^16^, but as well the structure of model compound **1**, we considered the synthesis of new derivatives having as *para* substituents at benzoyl rest from position 3 of indolizine: H, Cl, Br, Me and OMe. On the second phenyl ring, we chose as substituents in *para*: Me, OMe, F, Cl, Br and NO_2_ groups. In order to allow structure–activity relationship (SAR) comparisons with (*p*)substituted-benzoyl salts, we synthesized as well compounds having the OMe group as substituent in *meta*, and one compound having two OH groups in *meta* and *para* positions.

**Scheme 1.**
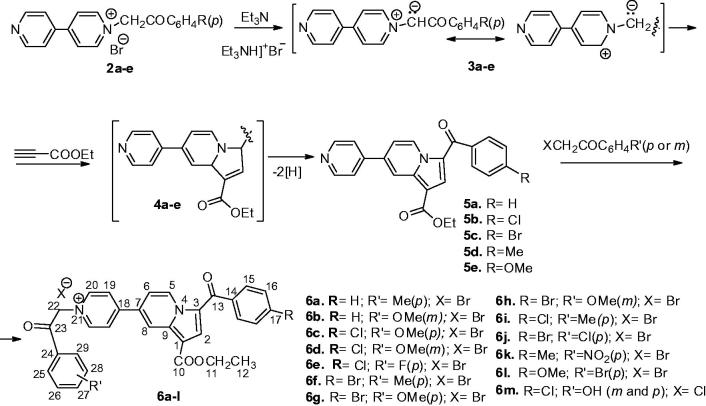
Synthesis pathway to obtain indolizineyl-pyridinium quaternary salts **6a**–**l**.

The synthesis of the new 4,4′-bipyridine derivatives was in line with the strategy reported previously by us^8^^,^^15^ and is presented in Scheme 1. Thus, 4,4′-bipyridine mono salts **2a**–**e** (obtained by 4,4′-bipyridine alkylation^17^) were used for the *in situ* generation of ylides **3a**–**e** which reacted with ethyl propiolate in [3 + 2] cycloaddition, leading to the intermediate compound **4a**–**e**, and finally to the completely aromatized monoindolizines **5a**–**e**. Another alkylation of indolizines **5a**–**e** using ω-bromoacetophenones led to the compounds **6a**–**m** (compounds **6a**–**h** and **6m** are new entities, while compounds **6i**–**l** were previously synthetized in our group^15^ (Scheme 1).

Alkylation towards **2a**–**e** is high yielding while cycloadditions led to indolizine **5a**–**e** in ∼50% yield. All compounds were fully characterised using elemental and spectral (NMR and IR) analysis.

### Activity against mycobacterium tuberculosis

**Figure 1.**
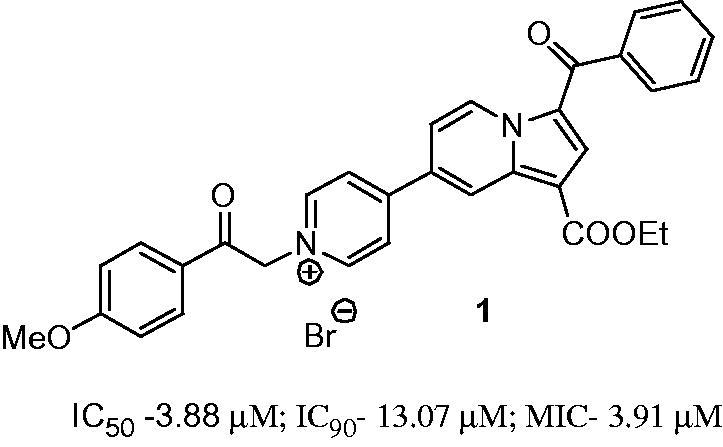
The structure of the reported compound 1 having anti-TB activity^8^.

The antimicrobial activity of compounds **6** against *Mycobacterium tuberculosis* H37Rv grown under aerobic conditions was evaluated as part of the TAACF TB screening program under direction of the US National Institute of Health, the NIAID division. The standard primary *in vitro* screen was assessed by determining the minimum inhibitory concentration at which growth was completely inhibited (MIC), and the concentrations that resulted in 50% and 90% inhibition of growth (IC_50_ and IC_90_ respectively)^18–21^. As can be seen in Table 1, eight compounds showed activity against *Mtb* H37Rv, five of them with a MIC <15 µM. Compound **6i** showed the best value of MIC, IC_50_ and IC_90_ from all tested compounds (Table 1), being superior to the model compound **1** (Figure 1).

**Table 1.** Results of antimycobacterial activity of compounds 6 against *M. tuberculosis* H37Rv grown under aerobic conditions.

Compound	MIC (μM)	IC_50_ (μM)	IC_90_ (μM)	Compound	MIC (μM)	IC_50_ (μM)	IC_90_ (μM)
**6a**	*14*	*13*	*15*	**6g**	50	35	46
**6b**	>50	39	>50	**6h**	*14*	*13*	*14*
**6c**	*15*	*12*	*14*	**6i**	***8***	***7.2***	***8.2***
**6d**	*15*	*14*	*16*	**6j**	>200	>200	>200
**6e**	>100	>100	>100	**6k**	>200	200	>200
**6f**	20	13	19	**6l**	>200	>200	>200
**6g**	50	35	46	**6m**	>100	>100	>100
Rifampicin	0.0065	0.0036	0.0071	Rifampicin	0.0065	0.0036	0.0071

Bold and italic values indicate the best ones from the series.

**Figure 2.**
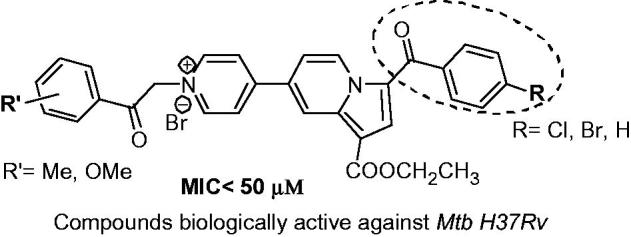
SAR conclusions (using anti-TB potential) of the current series.

Interestingly, the active compounds are only the ones having *para* substituent R= –Cl, –Br and –H and R’ = –Me(*para*) or –OMe(*para* or *meta*). Replacing *para* R group with –Me or –OMe and/or R’ group with –F(*p*), –Cl(*p*), –Br(*p*), –NO_2_(*p*) or –OH (*m* and *p*) led to a dropping of antimycobacterial activity (MIC >100 µM) (Figure 2). We thus hypothesised that the activity of these compounds bearing a *p-halogen-benzoyl* or a *benzoyl* moiety at position 3 of indolizine and a *p-methylbenzoyl or methoxy(p* or *m)benzoyl* moiety is somehow connected with a specifically interactions with putative binding sites.

Compounds **6a**, **6c**, **6d**, **6h** and **6i** that showed promising anti-TB activity in the primary assay, were subjected to the advanced antimycobacterial susceptibility profiling including MIC, IC_50_ and IC_90_ (repeated at lower starting concentrations), MIC under low oxygen, minimal bactericidal concentration (MBC), testing on drug-resistant *Mtb*, intracellular activity and cytotoxicity.

MIC, IC_50_ and IC_90_ determinations were repeated using similar assays for compounds **6a**, **6c**, **6d**, **6h** and **6i** and the obtained values (Table 2) were comparable with the previous ones (Table 1). The bactericidal activity of compounds was assessed against *Mtb H37Rv* grown in aerobic conditions. Viable cell counts are measured over 3 weeks of exposure to determine the rate of kill. MBC was defined as the minimum concentration required to achieve a 2-log kill in 21 d. For compounds with >1-log kill, an assessment of time- and/or concentration dependence was determined from the kill kinetics (DMSO was used as a positive control for growth). For compounds **6a**, **6c**, **6d** and **6i**, the effect of concentration predominates over that of time; therefore, these compounds display concentration-dependent effects that are significantly associated with an optimal free drug maximum concentration to MIC ratio^22^. For compound **6h** the effect of time is greater, displaying a time-dependent effect, and bacterial outcome is associated with free drug concentrations remaining above the MIC for a defined portion of the dosing interval^22^. The MIC value used in this experiment was taken from the first MIC assay presented herein. Encouraging, all five tested compounds are bactericidal against replicating cultures with MBCs equal or smaller then MICs.

**Table 2.** Revaluation of antimycobacterial activity of compounds **6a**, **6c**, **6d**, **6h** and **6i** against *M. tuberculosis* H37Rv grown under aerobic conditions.

Compound	MIC aerobic condition (μM)	IC_50_ (μM)	IC_90_ (μM)	Compound	MIC aerobic condition (μM)	IC_50_ (μM)	IC_90_ (μM)
**6a**	22	12	23	**6h**	27	15	26
**6c**	9.5	6.7	9.5	**6i**	16	9.8	17
**6d**	14	12	17	Rifampicin	0.0071	0.0043	0.0092

Traditional screening of drugs against *Mtb* only addressed or targets the organisms in an active replicating state. It is now widely accepted that *Mtb* can reside in a state of non-replicating persistence which has not been adequately assessed in the development of new antimicrobials. Therefore, we determined the antimycobacterial activity (MIC, IC_50_ and IC_90_) of the compounds **6** against *Mtb* H37Rv grown under hypoxic conditions using the low oxygen recovery assay (LORA)^23–25^. Bacteria are first adapted to low oxygen conditions and then exposed to compounds under hypoxia for 10 d followed by incubation under aerobic conditions (outgrowth) for 28 h. Parallel, oxygen-deprived bacteria were also inoculated into compound assay plates and incubated under aerobic conditions for 5 d. The growth in both assays was measured using luminescence (Table 3). Rifampicin was included in each plate and metronidazole was included in each run as positive controls for aerobic and anaerobic killing of *Mtb*, respectively^23–25^.

**Table 3.** The bactericidal activity (MBC) of compounds **6a**, **6c**, **6d**, **6h** and **6i**.

Compound	MIC (μM)	MBC (μM)	Concentration dependent	Time dependent
**6a**	14	14	Y	N
**6c**	15	3.75	Y	N
**6d**	15	3.75	Y	N
**6h**	14	14	N	Y
6i	8	8	Y	N

As can be seen in Table 4, all tested compounds showed a better antimycobacterial activity in anaerobic conditions than the control Metronidazole. Interestingly, for compounds **6a**, **6c**, **6d** and **6i**, MIC values in anaerobic conditions were smaller than the values obtained in aerobic conditions. Usually, the antimycobacterial agents targeting the cell wall are inactive in anaerobic conditions^26^; therefore, we presume that tested compounds **6** hit other cellular targets of *Mtb*.

**Table 4.** Results of antimycobacterial activity of compounds **6a**, **6c**, **6d**, **6h** and **6i** against *M. tuberculosis* H37Rv under low oxygen.

	Low oxygen			Normal oxygen		
Compound	MIC (μM)	IC_50_ (μM)	IC_90_ (μM)	MIC (μM)	IC_50_ (μM)	IC_90_ (μM)
**6a**	15	12	14	100	9.2	42
**6c**	57	6.5	18	75	8.1	**23**
**6d**	**41**	7.4	17	43	17	27
**6h**	100	7.9	37	**38**	16	24
**6i**	63	**1.9**	**10**	140	**6.5**	28
Rifampicin	0.018	0.0027	0.0067	0.021	0.0057	0.011
Metronidazole	200	30	64	>200	>200	>200

A good antimycobacterial activity of compounds **6a**, **6c**, **6d** and **6i** is maintained against five resistant isolates of *Mtb* strains under aerobic conditions^18–21^, especially for compounds **6c** and **6i** (see MIC, IC_50_ and IC_90_ values in Table 5). Strains tested were two isoniazid resistant strains (INH-R1 and INH-R2), two rifampicin resistant strains (RIF-R1 and RIF-R2) and a fluoroquinolone resistant strain (FQ-R1).

**Table 5.** MIC, IC_50_ and IC_90_ of compounds **6** against *M. tuberculosis* resistant at different treatments and non-tuberculous mycobacteria.

	INH-R1			INH-R2			RIF-R1			*M. avium*
Compound	MIC (μM)	IC_50_ (μM)	IC_90_ (μM)	MIC (μM)	IC_50_ (μM)	IC_90_ (μM)	MIC (μM)	IC_50_ (μM)	IC_90_ (μM)	MIC (μM)
**6a**	33	13	35	19	12	22	14	8.3	13	50
**6c**	**14**	**6.9**	**14**	**16**	**6.2**	**19**	**10**	**6.5**	**10**	50
**6d**	28	14	36	30	15	35	22	12	24	200
**6h**	>50	18	>50	>50	16	43	24	13	26	200
**6i**	**22**	**13**	**23**	**20**	**9.8**	**20**	**15**	**7.7**	**15**	50
**C1**	0.022	0.012	0.031	0.013	0.0070	0.016	5.3	1.1	3.8	0.098^3^
**C2**	>200	>200	>200	>200	>200	>200	0.092	0.063	0.094	–
**C3**	2.9	1.9	3.0	3.5	2.4	4.3	2.7	1.6	2.6	–
	RIF-R2			FQ-R1			*M. abscessus*			
	MIC (μM)	IC_50_ (μM)	IC_90_ (μM)	MIC (μM)	IC_50_ (μM)	IC_90_ (μM)	MIC (μM)	IC_50_ (μM)	IC_90_ (μM)	
**6a**	31	12	29	31	15	32	>200	24	37	
**6c**	**13**	**8.1**	**12**	**16**	**8**	**16**	**23**	16	21	
**6d**	16	12	14	32	21	32	>200	81	150	
**6h**	>50	15	>50	>50	26	>50	>200	>200	>200	
**6i**	**22**	**12**	**22**	**27**	**11**	**28**	**25**	14	23	
**C1**	>50	>50	>50	0.024	0.013	0.033	3.6	2.3	3.6	
**C2**	0.31	0.27	0.30	0.16	0.13	0.15	–	–	–	
**C3**	2.9	1.7	3.0	110	49	110	–	–	–	

INH: isoniazid-resistant strains; RIF: rifampicin-resistant strains; FQ: fluoroquinolone-resistant strains; C1: rifampicin control; C2: isoniazid control; C3: levofloxacin control.

Bold and italic values indicate the best ones from the series.

The antimycobacterial activity against nontuberculous mycobacteria (NTM) *Mycobacterium avium* and *Mycobacterium abscessus* was as well evaluated under aerobic conditions^18^^,^^21^^,^^27^. As can be seen in Table 5, only compounds **6c** and **6i** showed activity against *M. abscessus*, while compounds **6a**, **6c** and **6i** showed similar moderate activity (MIC = 50 µM) against *M. avium*.

The cytotoxixity of compounds towards eukaryotic cells was determined using the THP-1 human monocytic cell line, by calculating the concentration of compound causing 50% loss in viability (IC_50_)^27^ (Table 6). The cytotoxicity of tested compounds proved to be moderate to high, all compounds having a selectivity index (SI) < 1 (SI = IC_50_/MIC). These findings were somewhat disappointing, since structural elements of these compounds are part of different used drugs.

**Table 6.** Results of cytotoxicity evaluation.

Compound	Cytotoxicity IC_50_ (μM)	IC_50_ intracell (μM)	IC_90_ intracell (μM)
**6a**	2.9	5.2	15
**6c**	4.1	7	34
**6d**	3.9	11	>50
**6h**	3.8	13	43
**6i**	3.8	10	48
Control compound^a^^,^^b^	0.020^a^	0.18^b^	0.27^b^

a Staurosporine control.

b Isoniazid control.

Since the overall efficacy of any anti TB drug will be improved by its ability to traffic into the macrophage phagosome containing replicating bacteria, we evaluated the intracellular activity of compounds^24^. This was measured by using THP-1 cell line infected with *Mtb*. Infected cells were exposed to compounds for 72 h and viable bacterial counts were measured using luminescence as a measure of intracellular growth. The IC_50_ and IC_90_ were defined as the compounds concentrations that produced 50% and 90% inhibition of bacterial growth, respectively.

All tested compounds exhibited a good intracellular activity (IC_50_ = 7–13 µM), even if the results are inferior to the control Isoniazid (Table 6).

Taking into considerations the promising anti-TB activity of compound **6i**, a complete absorption, distribution, metabolism, excretion and toxicity (ADMET) study has been performed for it.

First, plasma protein binding (PPB) for compound **6i** was determined by equilibrium dialysis using a semi-permeable membrane which separates two compartments containing protein (human plasma) and buffer^28^^,^^29^. The experiments used propranolol as internal binding standard and warfarin as a high-binding control. Molecules can penetrate freely, but proteins cannot pass through the membrane. Compound **6i** was strongly bound to the plasma proteins (Table 7).

**Table 7.** Results of plasma protein binding assay for compound **6i**.

Compound	Test species	Mean plasma fraction unbound (%)	Mean plasma fraction bound (%)	Recovery
**6i**	Human	0.35	99.7	48.7
Propanolol	Human	20.7	79.3	93
Warfarin	Human	0.51	99.5	96.7

Usually high PPB is associated with a lower clearance rate resulting in a greater half-time *in vivo* compared with low protein binding compounds. Despite the fact that drugs with low protein binding are believed to be more efficacious because of higher free drug concentration, there are studies concluding that the binding of a drug to plasma proteins has little effect on the *in vivo* efficacy of that drug^29^^,^^30^.

The Caco-2 cell layer permeability assay is widely used as a more predictive *in vitro* model of absorption through the intestinal epithelium^31^. Therefore, the permeability (measured in both directions) of compound **6i** was assessed using a Caco-2-cell monolayer. For A–B permeability, compound **6i** was added to the apical side of the Caco-2 monolayer and the transport to the basal side monitored. For B–A permeability, test compound was added to the basal side of the Caco-2 monolayer and the transport of the compound to the apical side monitored. The amount of compound present in each compartment was quantified by LC-MS/MS. Each experiment included the control compounds atenolol (low permeability, paracellular transport), propranolol (high permeability, passive transcellular transport) and talinolol (P-gp efflux control)^32–36^ (Table 8).

**Table 8.** Permeability evaluation of compound **6i**.

Compound	Mean A→B P_app_^a^ (10^−6^cm/s)	Mean B→A P_app_^a^ (10^−6^cm/s)	Efflux ratio^b^
**6i**	0.76	1.3	1.7
Atenolol	0.13	0.37	2.8
Propanolol	12.7	25	2
Talinolol	0.077	3.3	43

a Papp is the apparent permeability rate coefficient = (dQ/dt)/(C_0_A) where dQ/dt is rate of permeation, C_0_ is the initial concentration of compound and A is the area of monolayer.

b Efflux ratio (R*e*) is P_app_ (B→A)/P_app_ (A→B). An R*e* > 2 indicated a potential substrate for P-glycoprotein or other active transporters.

Compound **6i** can be considered poorly permeable with a A→B P_app_ < 2, and shows a low active efflux (R*e* = 1.7). This led us to suppose that the mechanism of absorption of compound **6i** is almost a paracellular one with basically no involvement of transporter proteins. However, recent studies proved no significant correlation between antimycobacterial activity and Caco-2 permeability, indicating that permeability is not a predictor of activity inside of mycobacterium^31^.

Drug metabolism via the cytochrome P450 system has emerged as an important determinant in the occurrence of several drug-drug interactions that can result in drug toxicities, reduced pharmacological effect, and adverse drug reactions^37^. Therefore, compound **6i** was tested for inhibition of six cytochrome P450 enzyme isoforms: CYP2B6, CYP2C8, CYP2C9, CYP2C19, CYP2D6 and CYP3A4. For each assay, human liver microsomes are incubated with a probe substrate for each CYP isoform in the presence of compound. The formation of metabolites for each isoform was quantified by LC-MS/MS as a measure of enzyme activity^38–40^. For compound **6i**, enzyme activity was calculated and IC_50_ generated (Table 9).

**Table 9.** Cytochrome P450 inhibition results.

	IC_50_ (μM)						
Compound	CYP3A4-Midazolam	CYP3A4-Testosterone	CYP2C9	CYP2D6	CYP2C8	CYP2B6	CYP2C19
**6i**	0.48	0.081	>5	>5	>5	>5	>5
Ketoconazole^a^	0.033	0.022	–	–	–	–	–
Sulfaphenazole^a^	–	–	0.16	–	–	–	–
Quinidine^a^	–	–	–	0.032	–	–	–
Montelukast^a^	–	–	–	–	0.14	–	–
Tranylcypromine^a^	–	–	–	–	–	–	7.5
Ticlopidine^a^	–	–	–	–	–	0.72	–

a Control compounds.

Compound **6i** showed a high inhibition of CYP3A4-catalyzed testosterone and a moderate inhibition of midazolam 1’-hydroxylation, this profile suggesting a high potential for drug-drug interactions. No other CYPs were directly inhibited by **6i**, IC_50_s of **6i** on these CYPs being >5 µM.

Compound **6i** was tested for microsomal stability using pooled human liver S9 microsomes. Microsomes are incubated with the test compound at 37 °C in the presence of the co-factor NADPH; the reaction was terminated, the supernatant recovered and test compound quantified by LC-MS/MS. The stability of compound is expressed as a function of time^41^^,^^42^ (Table 10).

**Table 10.** *In vitro* microsomal stability assay.

Compound	C (μM)	Test species	NADPH-dependent CL_int_^a^ (μL/min/mg)	NADPH-dependent *T*_1/2_^b^ (min)	NADPH-free CL_int_^a^ (μL/min/mg)	NADPH-free *T*_1/2_^b^ (min)
**6i**	1	Human	369	6.3	<12.8	>180
Verapamil	1	Human	123	18.7	<12.8	>180
Dextromethorphan	1	Human	24.3	94.9	<12.8	>180

a Microsomal intrinsic clearance = ln(2)/(*T*_1/2_[microsomal protein]).

b Half-life = 0.693/−*k*, where *k* is the rate constant.

Compound **6i** proved to be a very highly cleared compound. Compounds with this profile are generally considered that they are likely to be rapidly cleared *in vivo* resulting in a short duration of action and, it should be cleared strongly *in vivo* by CYP metabolism^43^.

The cytotoxicity of compound **6i** was also tested towards eukaryotic cell using the human liver cells (HepG2), and Staurosporine as control (IC_50_= 0.0086 µM). The IC_50_ was determined as the concentration of compound causing a 50% loss of viability^44–47^. Compound **6i** showed an IC_50_ value of 7.0 µM, similar with its MIC value (8.0 µM), which maintains its cytotoxicity profile.

## Conclusion

In summary, we have employed the 4-(indolizine-7-yl)-pyridin-1-ium scaffold as core for the synthesis of 13 compounds in order to test their antimycobacterial activity. The reaction pathway is efficient and straight applicable, involving two N-alkylations of the 4,4,-bipyridine and, a Huisgen [3 + 2] dipolar cycloaddition of resulting ylides to ethyl propiolate. The primary antimycobacterial screening reveals that eight of the 13 tested compounds had a good activity against *Mycobacterium tuberculosis H37Rv* under aerobic conditions. SAR correlation reveals a certain influence of the R substituent from the *para* position of benzoyl moiety at position 3 of indolizine, the most active being compounds with R= –H, –Cl, –Br. The most active five compounds (namely **6a**, **6c**, **6d**, **6h**, **6i**) passed the second stage of anti TB testing, these including MIC, IC_50_ and IC_90_ (repeated at lower starting concentrations), MIC under low oxygen, MBC, testing on drug-resistant *Mtb* strains and nontuberculous mycobacteria, intracellular activity and cytotoxicity. These assay proved that our compounds are potent against both replicating and non-replicating *Mtb*, have a bactericidal mechanism of action, are active against drug-resistant *Mtb* strains, present a moderate to good activity against nontuberculous mycobacteria, a good intracellular activity, and a moderate to high cytotoxicity. The ADMET studies of compound **6i** show poor results, but motivating in the same time for further studies within the area of monoindolizine mono-salt.

## Supplementary Material

IENZ_1375483_Supplementary_Material.pdf

## References

[1] Dye C, Williams BG. The population dynamics and control of tuberculosis. Science 2010;328:856–61. 20466923 10.1126/science.1185449

[2] WHO. Global Tuberculosis Report. Geneva, Switzerland: WHO; 2016. Available from: http://www.who.int/tb/publications/global_report/en/ [last accessed 10 Oct 2017].

[3] Raviglione M, Marais B, Floyd K, et al Scaling up interventions to achieve global tuberculosis control: progress and new developments. Lancet 2012;397:1902–13. 10.1016/S0140-6736(12)60727-2 22608339

[4] Ma Z, Lienhardt C, Mclleron H, et al Global tuberculosis drug development pipeline: the need and the reality. Lancet 2010;5:1–10. 10.1016/S0140-6736(10)60359-9 20488518

[5] Crabb C. Global alliance at full steam for new TB drugs. Bull World Health Organ 2002;80:517. 12132014 PMC2567527

[6] Dulla B, Wan B, Franzblau SG, et al Construction and functionalization of fused pyridine ring leading to novel compounds as potential antitubercular agents. Bioorg Med Chem Lett 2012;22:4629–35. 22726932 10.1016/j.bmcl.2012.05.096

[7] Moraski GC, Markley LD, Chang M, et al Generation and exploration of new classes of antitubercular agents: the optimization of oxazolines, oxazoles, thiazolines, thiazoles to imidazo[1,2-a]pyridines and isomeric 5,6-fused scaffolds. Bioorg Med Chem 2012;20:2214–20. 22391032 10.1016/j.bmc.2012.02.025 PMC3304000

[8] Danac R, Mangalagiu II. Antimycobacterial activity of nitrogen heterocycles derivatives: bipyridine derivatives. Part III. Eur J Med Chem 2014;74:664–70. 24268596 10.1016/j.ejmech.2013.09.061

[9] Mantu D, Luca C, Moldoveanu C, et al Synthesis and antituberculosis activity of some new pyridazine derivatives. Part II. Eur J Med Chem 2010;45:5164–8. 20813433 10.1016/j.ejmech.2010.08.029

[10] Al Matarneh CM, Ciobanu CI, Mangalagiu II, et al Design, synthesis and antimycobacterial evaluation of some new azaheterocycles with 4,7-phenanthroline skeleton. Part VI. J Serb Chem Soc 2016;81:133–40.

[11] Danac R, Al Matarneh CM, Shova S, et al New indolizinees with phenanthroline skeleton: synthesis, structure, antimycobacterial and anticancer evaluation. Bioorg Med Chem 2015;23:2318–27. 25882524 10.1016/j.bmc.2015.03.077

[12] Danac R, Daniloaia T, Antoci V, et al Design, synthesis and antimycobacterial activity of some new azaheterocycles: phenanthroline with *p-halo*-benzoyl Skeleton. Part V. Lett Drug Des Discov 2015;12:14–17.

[13] Butnariu RM, Mangalagiu II. New pyridazine derivatives: synthesis, chemistry and biological activity. Bioorg Med Chem 2009;17:2823–9. 19272782 10.1016/j.bmc.2009.02.028

[14] Caprosu M, Butnariu R, Mangalagiu II. Synthesis and antimicrobial activity of some new pyridazine derivatives. Heterocycles 2005;65:1871–9.

[15] Rotaru A, Druta I, Avram E, et al Synthesis and properties of fluorescent 1,3-substituted mono and biindolizines. Arkivoc 2009;13:287–99.

[16] Dholariya HR, Patel KS, Patel JC, et al Dicoumarol complexes of Cu (II) based on 1, 10-phenanthroline: synthesis, X-ray diffraction studies, thermal behaviour and biological evaluation. Spectrochim Acta A 2013;108:319–28. 10.1016/j.saa.2012.09.096 23478077

[17] Rotaru A, Danac R, Druta I, et al The synthesis and the biological activity of diquaternary salts derivatives of 4,4`-bipyridyl. Rev Chim (Bucharest) 2005;56:179–83.

[18] Ollinger J, Bailey MA, Moraski GC, et al A dual read-out assay to evaluate the potency of compounds active against *Mycobacterium tuberculosis*. PLoS One 2013;8:e60531. 23593234 10.1371/journal.pone.0060531 PMC3617142

[19] Zelmer A, Carroll P, Andreu N, et al A new *in vivo* model to test anti-tuberculosis drugs using fluorescence imaging. J Antimicrob Chemother 2012;67:1948–60. 22635525 10.1093/jac/dks161 PMC3394442

[20] Carroll P, Schreuder LJ, Muwanguzi-Karugaba J, et al Sensitive detection of gene expression in mycobacteria under replicating and non-replicating conditions using optimized far-red reporters. PLoS One 2010;5:e9823. 20352111 10.1371/journal.pone.0009823 PMC2843721

[21] Lambert RJ, Pearson J. Susceptibility testing: accurate and reproducible minimum inhibitory concentration (MIC) and non-inhibitory concentration (NIC) values. J Appl Microbiol 2000;88:788–90. 10.1046/j.1365-2672.2000.01017.x 10792538

[22] Kuti JL. Optimizing Antimicrobial Pharmacodynamics: a guide for stewardship program. Rev Med Clin Condes 2016;27:615–24.

[23] Cho SH, Warit S, Wan B, et al Low-oxygen-recovery assay for high-throughput screening of compounds against nonreplicating *Mycobacterium tuberculosis*. Antimicrob Agents Chemother 2007;51:1380–5. 17210775 10.1128/AAC.00055-06 PMC1855511

[24] Andreu N, Zelmer A, Fletcher T, et al Optimisation of bioluminescent reporters for use with mycobacteria. PLoS One 2010;5:e10777. 20520722 10.1371/journal.pone.0010777 PMC2875389

[25] Wayne LG. *In vitro* model of hypoxically induced nonreplicating persistence of *Mycobacterium tuberculosis* In: Parish T, Stoker NG, eds. Mycobacterium tuberculosis protocols. Totowa, NJ: Humana Press; 2001:247–270. 10.1385/1-59259-147-7:247 21341080

[26] Favrot L, Ronning DR. Targeting the mycobacterial envelope for tuberculosis drug development. Expert Rev Anti Infect Ther 2012;10:1023–36. 23106277 10.1586/eri.12.91 PMC3571691

[27] Franzblau SG, Witzig RS, McLaughlin JC, et al Rapid, low-technology MIC determination with clinical *Mycobacterium tuberculosis* isolates by using the microplate Alamar Blue Assay. J Clin Microbiol 1998;36:362–6. 9466742 10.1128/jcm.36.2.362-366.1998 PMC104543

[28] Banker MJ, Clark TH, Williams JA. Development and validation of a 96-well equilibrium dialysis apparatus for measuring plasma protein binding. J Pharm Sci 2003;92:967–74. 12712416 10.1002/jps.10332

[29] Smith DA, Di L, Kerns EH. The effect of plasma protein binding on in vivo efficacy: misconceptions in drug discovery. Nat Rev Drug Discov 2010;9:929–39. 21119731 10.1038/nrd3287

[30] Ramesh R, Shingare RD, Kumar V, et al Repurposing of a drug scaffold: Identification of novel sila analogues of rimonabant as potent antitubercular agents. Eur J Med Chem 2016;122:723–30. 27476117 10.1016/j.ejmech.2016.07.009

[31] Lakshminarayana SB, Huat TB, Ho PC, et al Comprehensive physicochemical, pharmacokinetic and activity profiling of anti-TB agents. J Antimicrob Chemother 2015;70:857–67. 25587994 10.1093/jac/dku457 PMC7714050

[32] Stewart BH, Chan OH, Lu RH, et al Comparison of intestinal permeabilities determined in multiple *in vitro* and *in situ* models: relationship to absorption in humans. Pharm Res 1995;12:693–9. 7479555 10.1023/a:1016207525186

[33] Artursson P, Palm K, Luthman K. Caco-2 monolayers in experimental and theoretical predictions of drug transport. Adv Drug Deliv Rev 2001;46:27–43. 11259831 10.1016/s0169-409x(00)00128-9

[34] Yee S In vitro permeability across Caco-2 cells (colonic) can predict in vivo (small intestinal) absorption in man-fact or myth. Pharm Res 1997;14:763–6. 9210194 10.1023/a:1012102522787

[35] Endres CJ, Hsiao P, Chung FS, et al The role of transporters in drug interactions. Eur J Pharm Sci 2006;27:501–17. 16364611 10.1016/j.ejps.2005.11.002

[36] Balimane PV, Han YH, Chong S. Current industrial practices of assessing permeability and P-glycoprotein interaction. APS J 2006;8:E1–E13. 10.1208/aapsj080101 PMC2751418 16584115

[37] Ogu CC, Maxa JL. Drug interactions due to cytochrome P450. *Proc (Bayl Univ Med Cent) 2000;13:421–3. 16389357 10.1080/08998280.2000.11927719 PMC1312247

[38] Kim MJ, Kim H, Cha IJ, et al High-throughput screening of inhibitory potential of nine cytochrome P450 enzymes in vitro using liquid chromatography/tandem mass spectrometry. Rapid Commun Mass Spectrom 2005;19:2651–8. 16124035 10.1002/rcm.2110

[39] Walsky RL, Obach RS. Validated assays for human cytochrome P450 activities. Drug Metab Dispos 2004;32:647–60. 15155557 10.1124/dmd.32.6.647

[40] Fowler S, Zhang H. *In vitro* evaluation of reversible and irreversible cytochrome P450 inhibition: current status on methodologies and their utility for predicting drug–drug interactions. AAPS J 2008;10:410–24. 18686042 10.1208/s12248-008-9042-7 PMC2751392

[41] Houston JB. Utility of in vitro drug metabolism data in predicting in vivo metabolic clearance. Biochem Pharmacol 1994;47:1469–79. 8185657 10.1016/0006-2952(94)90520-7

[42] Obach RS. Prediction of human clearance of twenty-nine drugs from hepatic microsomal intrinsic clearance data: an examination of in vitro half-life approach and non-specific binding to microsomes. Drug Metab Dispos 1999;27:1350–9. 10534321

[43] Di L, Kerns EH, Ma XJ, et al Applications of high throughput microsomal stability assay in drug discovery. Comb Chem High Throughput Screen 2008;11:469–76. 18673274 10.2174/138620708784911429

[44] Crouch SP, Kozlowski R, Slater KJ, et al The use of ATP bioluminescence as a measure of cell proliferation and cytotoxicity. J Immunol Methods 1993;160:81–8. 7680699 10.1016/0022-1759(93)90011-u

[45] Lundin A, Hasenson M, Persson J, et al Estimation of biomass in growing cell lines by adenosine triphosphate assay. Meth Enzymol 1986;133:27–42. 3821540 10.1016/0076-6879(86)33053-2

[46] Maehara Y, Anai H, Tamada R, et al The ATP assay is more sensitive than the succinate dehydrogenase inhibition test for predicting cell viability. Eur J Cancer Clin Oncol 1987;23:273–6. 3109921 10.1016/0277-5379(87)90070-8

[47] Slater K. Cytotoxicity tests for high-throughput drug discovery. Curr Opin Biotechnol 2001;12:70–4. 11167076 10.1016/s0958-1669(00)00177-4

