# Supplementary material for: Antimycobacterial activity of nitrogen heterocycles derivatives: 7-(pyridine-4-yl)-indolizine derivatives. Part VII8–12
Source: J Enzyme Inhib Med Chem. 2017 Oct 26;32(1):1291–8. doi: 10.1080/14756366.2017.1375483 (PMC6021032; doi:10.1080/14756366.2017.1375483)
Supplement: IENZ_1375483_Supplementary_Material.pdf [file IENZ_A_1375483_SM5319.pdf]

Appendix

Antimycobacterial activity of nitrogen heterocycles derivatives: 7-(pyridine-4-yl)-indolizine derivatives. Part VII[8-12]

Anda-Mihaela Olaru[a], Violeta Vasilache[b], Ramona Danac[a*], Ionel I. Mangalagiu[a*]

a. "Alexandru Ioan Cuza" University of Iasi, Faculty of Chemistry, 11 Carol I, Iasi 700506, Romania

b. "Alexandru Ioan Cuza" University of Iasi, CERNESIM Research Centre, 11 Carol I, Iasi 700506, Romania

[*]Corresponding author

Tel.:+40 232 201343; fax: +40 232 201313.

E-mail addresses: rdanac@uaic.ro (R. Danac); ionelm@uaic.ro (I. I. Mangalagiu)

Protocols concerning the antimycobacterial evaluation of tested compounds:

1. The primary cycle high throughput screening (HTS) - Cycle 1 determination of 90% inhibitory concentration (IC_{90}), 50% inhibitory concentration (IC_{50}) and Minimum Inhibitory Concentration (MIC) under Aerobic Conditions

The MIC of compound was determined by measuring bacterial growth after 5 d in the presence of test compounds. Compounds were prepared as 10-point two-fold serial dilutions in DMSO and diluted into 7H9-Tw-OADC medium in 96-well plates with a final DMSO concentration of 2%. The highest concentration of compound was 200 µM where compounds were soluble in DMSO at 10 mM. For compounds with limited solubility, the highest concentration was 50X less than the stock concentration e.g. 100 µM for 5 mM DMSO stock, 20 µM for 1 mM DMSO stock. Each plate included assay controls for background (medium/DMSO only, no bacterial cells), zero growth (100 µM rifampicin) and maximum growth (DMSO only), as well as a rifampicin dose response curve. Plates were inoculated with *M. tuberculosis* and incubated for 5 days: growth was measured by OD_{590} and

fluorescence (Ex 560/Em 590) using a BioTek™ Synergy 4 plate reader. Growth was calculated separately for OD_{590} and RFU. To calculate the MIC, the 10-point dose response curve was plotted as % growth and fitted to the Gompertz model using GraphPad Prism 5. The MIC was defined as the minimum concentration at which growth was completely inhibited and was calculated from the inflection point of the fitted curve to the lower asymptote (zero growth) (Figure 1A). In addition dose response curves were generated using the Levenberg-Marquardt algorithm and the concentrations that resulted in 50% and 90% inhibition of growth were determined (IC_{50} and IC_{90} respectively) (Figure 1B). Raw data is provided and can be used to plot either type of curve [1-4].

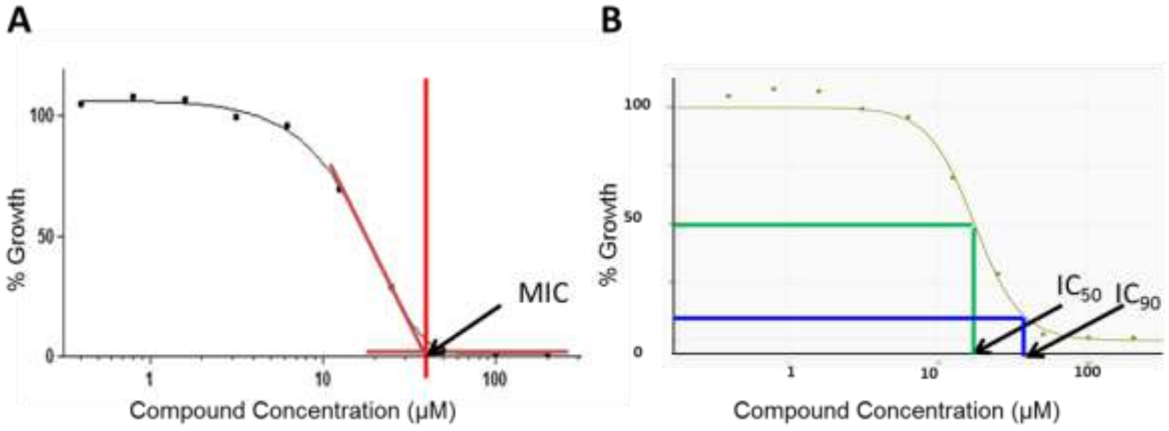

Figure 1. Dose response curves used to calculate MIC, IC_{50} and IC_{90}. Data points obtained from a dose response growth inhibition assay are curve-fitted using **(A)** the Gompertz model to calculate MIC and **(B)** the Levenberg-Marquardt algorithm to calculate IC_{50} and IC_{90}. **(A)** The MIC is the concentration at which complete inhibition of growth is seen and is derived from the point of inflection at which the curve meets the lower asymptote (zero growth). **(B)** IC_{50} and IC_{90} are points at which growth is inhibited by 50% and 90% respectively. Orange line = MIC; Green line = IC_{50}; Blue line = IC_{90}.

2. *Cycle 2 - In vitro* assessments for antimicrobial activity

2.1. Minimum Inhibitory Concentration (MIC)

The MIC of compound was determined by measuring bacterial growth after 5 d in the presence of test compounds. Compounds were prepared as 20-point two-fold serial dilutions in DMSO and diluted into 7H9-Tw-OADC medium in 96-well plates with a final DMSO concentration of 2%. The highest concentration of compound was 200 μM where compounds were soluble in DMSO at 10 mM. Each plate included assay controls for background

(medium/DMSO only, no bacterial cells), zero growth (100 µM rifampicin) and maximum growth (DMSO only), as well as a rifampicin dose response curve. Plates were inoculated with M. tuberculosis and incubated for 5 days: growth was measured by OD_{590} and fluorescence (Ex 560/Em 590) using a BioTek™ Synergy 4 plate reader. Growth was calculated separately for OD_{590} and RFU. To calculate the MIC, the dose response curve was plotted as % growth and fitted to the Gompertz model using GraphPad Prism 5. The MIC was defined as the minimum concentration at which growth was completely inhibited and was calculated from the inflection point of the fitted curve to the lower asymptote (zero growth) (Figure 1A). In addition dose response curves were generated using the Levenberg-Marquardt algorithm and the concentrations that resulted in 50% [1-4].

2.2. Minimal Bactericidal Concentration (MBC)

M. tuberculosis was grown aerobically to logarithmic phase and inoculated into liquid medium containing four different compound concentrations with a final maximum concentration of 2% DMSO. For compounds with MIC < 20 µM (from Task Group 1 assay), the concentration selected were 10X MIC, 5X MIC, 1X MIC and 0.25XMIC. For compounds with MIC > 20 µM, the highest concentration possible was tested (200, 100, 20 and 5 µM). Cultures were exposed to compounds for 21 days and cell viability measured by enumerating colony forming units on agar plates on day 0, 7, 14 and 21.

MBC was defined as the minimum concentration required to achieve a 2-log kill in 21 days. For compounds with >1-log kill, an assessment of time- and/or concentration-dependence was determined from the kill kinetics. DMSO was used as a positive control for growth.

2.3. Low-Oxygen Recovery Assay (LORA)

Test compounds were prepared as 20-point two-fold serial dilutions in DMSO and diluted into DTA medium in 96-well plates with a final DMSO concentration of 2%. The highest concentration of compound was 200 µM where compounds were soluble in DMSO at 10 mM. Control compounds were prepared as two-fold serial dilutions in DMSO and diluted into DTA medium in 96-well plates with a final DMSO concentration of 2%.

M. tuberculosis constitutively expressing the luxABCDE operon was inoculated into DTA medium in gas-impermeable glass tubes and incubated for 18 days to generate hypoxic

conditions (Wayne model of hypoxia). At this point, bacteria are in a non-replicating state (NRP stage 2) induced by oxygen depletion.

Oxygen-deprived bacteria were inoculated into compound assay plates and incubated under anaerobic conditions for 10 days followed by incubation under aerobic conditions (outgrowth) for 28h. Growth was measured by luminescence. Oxygen-deprived bacteria were also inoculated into compound assay plates and incubated under aerobic conditions for 5 days. Growth was measured by luminescence. Rifampicin was included in each plate and metronidazole was included in each run as positive controls for aerobic and anaerobic killing of *M. tuberculosis*, respectively [5-7].

2.4. MIC, IC_{50} and IC_{90} of compounds against M. Tuberculosis resistant at different treatment and nontuberculous mycobacteria

The MIC of compound was determined by measuring bacterial growth after 5 d in the presence of test compounds. Compounds were prepared as 10-point two-fold serial dilutions in DMSO and diluted into 7H9-Tw-OADC medium in 96-well plates with a final DMSO concentration of 2%. The highest concentration of compound was 200 µM where compounds were soluble in DMSO at 10 mM. Each plate included assay controls for background (medium/DMSO only, no bacterial cells), zero growth (100 µM rifampicin) and maximum growth (DMSO only), as well as a rifampicin dose response curve. Plates were inoculated with *M. tuberculosis* and incubated for 5 days; growth was measured by OD_{590}. To calculate the MIC, the 10-point dose response curve was plotted as % growth and fitted to the Gompertz model using GraphPad Prism 5. The MIC was defined as the minimum concentration at which growth was completely inhibited and was calculated from the inflection point of the fitted curve to the lower asymptote (zero growth). In addition dose response curves were generated using the Levenberg-Marquardt algorithm and the concentrations that resulted in 50% and 90% inhibition of growth were determined (IC_{50} and IC_{90} respectively). Raw data is provided and can be used to plot either type of curve [1-4].

2.5. MIC Against Other Disease-relevant Mycobacteria

The MIC of compound was determined by measuring bacterial growth in the presence of test compounds. Compounds were prepared as 20-point two-fold serial dilutions in DMSO and diluted into 7H9-Tw-OADC medium in 96-well plates with a final DMSO concentration

of 2%. The highest concentration of compound was 200 µM where compounds were soluble in DMSO at 10 mM. Each plate included assay controls for background (medium/DMSO only, no bacterial cells), zero growth (100 µM rifampicin) and maximum growth (DMSO only), as well as a rifampicin dose response curve.

Mycobacterium abscessus

Plates were inoculated with M. abscessus and incubated for 3 days at 37˚C; growth was measured by OD_{590}. To dose response curve was plotted as % growth and fitted to the Gompertz model. The MIC was defined as the minimum concentration at which growth was completely inhibited and was calculated from the inflection point of the fitted curve to the lower asymptote (zero growth). In addition dose response curves were generated using the Levenberg-Marquardt algorithm and the concentrations that resulted in 50% and 90% inhibition of growth were determined (IC_{50} and IC_{90} respectively). Raw data is provided and can be used to plot either type of curve. Rifampicin was included once in each run.

Mycobacterium avium

Plates were inoculated with M. avium, incubated for 5 days at 37˚C and Alamar blue was added to each well (10 µL of Alamar blue to 100 µL culture) and incubated for 24 h at 37˚C. Plates were visually inspected and the color recorded for each well. MIC was defined as the lowest concentration at which no metabolic activity was seen (blue well). [1,4,8]

2.6. Intracellular Activity and Cytotoxicity

The cytotoxicity of compounds was determined by measuring THP-1 cell viability after 3 days in the presence of test compounds. Compounds were prepared as 10-point three-fold serial dilutions in DMSO. The highest concentration of compound tested was 50 µM where compounds were soluble in DMSO at 10 mM. Each plate included staurosporine as a control. THP-1 cells were cultured in complete RPMI and differentiated into macrophage-like cells using 80 nM PMA overnight at 37°C, 5% CO_2. Cells were inoculated into assay plates and cultured for 24h before compound dilutions were added to a final DMSO concentration of 0.5%. Each run included staurosporine as a control. Assay plates were incubated for 3 days at 37°C, 5% CO_2; growth was measured using the CellTiter-Glo® Luminescent Cell Viability

Assay (Promega) which uses ATP as an indicator of cell viability. Relative luminescent units (RLU) were measured using a Biotek Synergy 4 plate reader. The dose response curve was fitted using the Levenberg–Marquardt algorithm. The IC_{50} was defined as the compound concentration that produced 50% inhibition of growth.

Intracellular Activity Assay

The activity of compounds against intracellular bacteria was determined by measuring viability in infected THP-1 cell after 3 days in the presence of test compounds. Compounds were prepared as 10-point three-fold serial dilutions in DMSO. The highest concentration of compound tested was 50 µM where compounds were soluble in DMSO at 10 mM.

THP-1 cells were cultured in complete RPMI and differentiated into macrophage-like cells using 80 nM PMA overnight at 37°C, 5% CO_2. THP-1 cells were infected with a luminescent strain of H37Rv (which constitutively expresses luxABCDE) at a multiplicity of infection of 1 and incubated overnight at 37°C, 5% CO_2. Infected cells were recovered using Accutase/EDTA solution, washed twice with PBS to remove extracellular bacteria and seeded into assay pates. Compound dilutions were added to a final DMSO concentration of 0.5%. Assay plates were incubated for 72 h at 37°C, 5% CO_2. Each run included isoniazid as a control. Relative luminescent units (RLU) were measured using a Biotek Synergy 2 plate reader. The dose response curve was fitted using the Levenberg–Marquardt algorithm. The IC_{50} and IC_{90} were defined as the compound concentrations that produced 50% and 90 % inhibition of growth respectively. [6]

3. *Cycle 3 – Absorption, distribution, metabolism, excretion and toxicity (ADMET) studies of compound 6i*

3.1. Plasma Protein Binding Assay

Compounds were added to human plasma at a fixed concentration of 5 µM. The mixture was dialyzed in a RED device (Rapid Equilibrium Dialysis, Pierce) against PBS and incubated on an orbital shaker for 4 h at 37˚C. Aliquots from plasma and PBS sides were collected; an equal volume of PBS was added to the plasma sample, and an equal volume of plasma was added to the PBS sample. Three volumes of methanol (containing the internal binding standard propranolol) was added to precipitate the proteins and release the compound. Each compound was tested in duplicate. Samples were centrifuged, the supernatant was

recovered and analyzed by LC-MS/MS. Each experiment included warfarin as a high-binding control. [9,10]

3.2. *Caco-2 Permeability Assay*

Caco-2 cells were trypsinized, resuspended in medium, and dispensed into a Millipore 96-well Caco-2 plate. The cells were allowed to grow and differentiate for three weeks, with feeding at 2-day intervals. For Apical to Basolateral (A→B) permeability, the compound was added to the apical (A) side and amount of permeation was determined on the basolateral (B) side; for Basolateral to Apical (B→A) permeability, the compound was added to the B side and the amount of permeation was determined on the A side. Each experiment included the control compounds atenolol (low permeability), propranolol (high permeability) and talinolol (P-gp efflux control).

Quality control

The A-side contained 100 μM Lucifer yellow in transport buffer pH 6.5, and the B-side contained transport buffer at pH 7.4. Caco-2 cells were incubated with these buffers for either 1h or 2 h, and the receiver side buffer was removed for analysis by LC-MS/MS. Aliquots of the cell buffers were analyzed by fluorescence to determine the transport of the impermeable dye Lucifer yellow to verify the Caco-2 cell monolayers were properly formed. Any deviations from control values were reported. [11-15]

3.3. *Cytochrome P450 Inhibition Assay*

Compounds were prepared as a 7-point dilution series in acetonitrile:DMSO (9:1). The final DMSO content in the reaction mixture was equal in all solutions used within an assay, and was < 0.2%. Samples were run in duplicate. Compounds were incubated with human liver microsomes in sample buffer containing 2 mM NADPH and probe substrate in a 200 μL assay final volume. Reactions were incubated at 37°C for the optimal time (10-60 min) and terminated by addition of methanol containing internal standard (propranolol) for analytical quantification. Samples were incubated at 4°C for 10 min and centrifuged at 4°C for 10 min. The supernatant was removed and the probe substrate metabolite was analyzed by LC-

MS/MS. A decrease in the formation of the metabolite compared to vehicle control was used to calculate an IC_{50} value (the test concentration which produces 50% inhibition). [16-18]

3.4. *In vitro Microsomal Stability Assay*

Compounds were incubated with human liver microsomes at 37ºC in duplicate. Each reaction contained 0.3 mg/mL human microsomal protein in assay buffer. Samples were removed at 0, 5, 15, 30, and 45 minutes, mixed with an equal volume of stop solution (containing propranolol as an internal standard), and incubated for >10 min at -20ºC. An additional volume of water was added, samples were centrifuged to remove precipitated protein and the supernatants were analyzed by LC-MS/MS to quantitate the remaining parent compound. A control reaction omitting NADPH (control buffer) was performed for each compound to detect NADPH-free degradation. Verapamil and dextromethorphan were included as control compounds. [19,20]

Data analysis

Data were converted to % compound remaining compared to the time zero. Data were fitted to a first order decay model to determine the compound half-life. Intrinsic clearance (CLint) was calculated from the half-life and the protein concentrations:

CL_{int} = ln(2)/($T_{1/2}$ [microsomal protein])

$T_{1/2}$ = 0.693/-k

CL_{int} = intrinsic clearance; $T_{1/2}$ = half-life; k = slope

3.5. *HepG2 Cytotoxicity*

The cytotoxicity of compounds was determined by measuring HepG2 cell viability growth after 3 d in the presence of test compounds. Compounds were prepared as 10-point three-fold serial dilutions in DMSO. The highest concentration of compound tested was 100 µM where compounds were soluble in DMSO at 10 mM. HepG2 cells were cultured in complete DMEM, inoculated into 384-well assay plates containing compounds and incubated for 24 h at 37ºC, 5% CO_2. Compounds were added and cells were cultured for a further 72 h.

The final DMSO concentration was 1%. Cell viability was determined using the CellTiter-Glo® Luminescent Cell Viability Assay (Promega) and measuring relative luminescent units (RLU). The dose response curve was fitted using the Levenberg–Marquardt algorithm. The TC_{50} was defined as the compound concentration that produced 50% loss of cell viability. Each run included staurosporine as a control. [21-24]

References

[1] J. Ollinger, M.A. Bailey, G.C. Moraski, A. Casey, S. Florio, T. Alling, M.J. Miller, T. Parish, A dual read-out assay to evaluate the potency of compounds active against *Mycobacterium tuberculosis*, PLOS One 8 (2013) e60531.

[2] A. Zelmer, P. Carroll, N. Andreu, K. Hagens, J. Mahlo, N. Redinger, B.D. Robertson, S. Wiles, T.H. Ward, T. Parish, J. Ripoll, G.J. Bancroft, U.E. Schaible, A new *in vivo* model to test anti-tuberculosis drugs using fluorescent imaging, J. Antimicrob. Chemother. 67 (2012) 1948-1960.

[3] P. Carroll, L.J. Schreuder, J. Muwanguzi-Karugaba, S. Wiles, B.D. Robertson, J. Ripoll, T.H. Ward, G.J. Bancroft, U.E. Schaible, T. Parish, Sensitive detection of gene expression in mycobacteria under replicating and non-replicating conditions using optimized far-red reporters, PLoS One 5 (2010) e9823.

[4] R.J. Lambert and J. Pearson, Susceptibility testing: accurate and reproducible minimum inhibitory concentration (MIC) and non-inhibitory concentration (NIC) values, J. Appl. Microbiol. 88 (2000) 788-790.

[5] S.H. Cho, S. Warit, B. Wan, C.H. Hwang, G.F. Pauli, S.G. Franzblau, Low-oxygen-recovery assay for high-throughput screening of compounds against nonreplicating *Mycobacterium tuberculosis*, Antimicrob. Ag. Chemother. 51 (2007) 1380-1385.

[6] N. Andreu, A. Zelmer, T. Fletcher, P.T. Elkington, T.H. Ward, J. Ripoll, T. Parish, G.J. Bancroft, U. Schaible, B.D. Robertson, S. Wiles, Optimisation of bioluminescent reporters for use with mycobacteria, PLoS One 5 (2010) e10777.

[7] L.G. Wayne, *In vitro* model of hypoxically induced nonreplicating persistence of *Mycobacterium tuberculosis*, in: T. Parish, N.G. Stoker (Eds), Mycobacterium tuberculosis protocols: Humana Press, 2001, pp. 247-270.

[8] S.G. Franzblau, R.S. Witzig, J.C. McLaughlin, P. Torres, G. Madico, A. Hernandez, M.T. Degnan, M.B. Cook, V.K. Quenzer, R.M. Ferguson, R.H. Gilman Rapid, low-technology MIC determination with clinical Mycobacterium tuberculosis isolates by using the microplate Alamar Blue Assay, J. Clin. Microbiol. 36 (1998) 362-366.

[9] M.J. Banker, T.H. Clark, J.A. Williams, Development and validation of a 96-well equilibrium dialysis apparatus for measuring plasma protein binding, J. Pharm. Sci. 92 (2003) 967-974.

[10] D.A. Smith, L. Di, E.H. Kerns, The effect of plasma protein binding on in vivo efficacy: misconceptions in drug discovery, Nat. Rev. Drug Discov. 9 (2010) 929-939.

[11] B.H. Stewart, O.H. Chan, R.H. Lu, E.L. Reyner, H.L. Schmid, H.W. Hamilton, B.A. Steinbuagh, M.D. Taylor, Comparison of intestinal permeabilities determined in multiple in vitro and in situ models: relationship to absorption in humans, Pharm. Res. 12 (1995) 693-699.

[12] P. Artursson, K. Palm, K. Luthman, Caco-2 monolayers in experimental and theoretical predictions of drug transport, Adv. Drug Deliv Rev. 46 (2001) 27-43.

[13] S. Yee, In vitro permeability across Caco-2 cells (colonic) can predict in vivo (small intestinal) absorption in man – fact or myth, Pharm. Res. 14 (1997) 763-766.

[14] C.J. Endres, P. Hsiao, F.S. Chung, J.D. Unadkat, The role of transporters in drug interactions, Eur. J. Pharm. Sci. 27 (2006) 501-517.

[15] P.V. Balimane, Y.H. Han, S. Chong, Current industrial practices of assessing permeability and P-glycoprotein interaction, AAPS J. 8 (2006) E1-E13.

[16] M.J. Kim, H. Kim, I.J. Cha, J.H. Shon, K.H. Liu, J.G. Shin, High-throughput screening of inhibitory potential of nine cytochrome P450 enzymes in vitro using liquid chromatography/tandem mass spectrometry, Rapid Commun. Mass Spectrom. 19 (2005) 2651-2658.

[17] R.L. Walsky, R.S. Obach, Validated Assays for Human Cytochrome P450 Activities. Drug Metab. Dispos. 32 (2004) 647-660.

[18] S. Fowler, H. Zhang, In vitro evaluation of reversible and irreversible cytochrome P450 inhibition: current status on methodologies and their utility for predicting drug–drug interactions, AAPS J. 10 (2008) 410-424.

[19] J.B. Houston, Utility of in vitro drug metabolism data in predicting in vivo metabolic clearance, Biochem. Pharmacol. 47 (1994) 1469-1479.

[20] R.S. Obach, Prediction of human clearance of twenty-nine drugs from hepatic microsomal intrinsic clearance data: an examination of in vitro half-life approach and non-specific binding to microsomes, Drug Metab. Dispos. 27 (1999) 1350-1359.

[21] S.P. Crouch, R. Kozlowski, K.J. Slater, J. Fletcher, The use of ATP bioluminescence as a measure of cell proliferation and cytotoxicity, J. Immunol. Methods. 160 (1993) 81-88.

[22] A. Lundin, M. Hasenson, J. Persson, A. Pousette, Estimation of biomass in growing cell lines by ATP assay, Methods Enzymol. 133 (1986) 27-42.

[23] Y. Maehara, H. Anai, R. Tamada, K. Sugimachi, The ATP assay is more sensitive than the succinate dehydrogenase inhibition test for predicting cell viability, Eur. J. Cancer Clin. Oncol. 23 (1987) 273-276.

[24] K. Slater, Cytotoxicity tests for high-throughput drug discovery, Curr. Opin. Biotechnol. 12 (2001) 70-74.
